# The 2024 international society for hip preservation physiotherapy agreement on post-operative rehabilitation following hip arthroscopy for femoroacetabular impingement syndrome

**DOI:** 10.1093/jhps/hnaf055

**Published:** 2025-10-27

**Authors:** Ashley Disantis, Nicolas Bonin, Thomas Byrd, Ashley Campbell, John Christoforetti, Michael Dienst, Thomas Ellis, Keelan Enseki, Benjamin Kivlan, David Kohlrieser, Liran Lifshitz, Robert Manske, Michael McClincy, John O’Donnell, Marc Safran, Amir Takla, Timothy Tyler, Michael Voight, RobRoy Martin

**Affiliations:** Department of Physical Therapy, UPMC Children’s Hospital of Pittsburgh, 4401 Penn Ave, Pittsburgh, PA 15224, USA; Duquesne University, Department of Physical Therapy, 141 Health Sciences Building, 600 Forbes Avenue, Pittsburgh, PA 15282, USA; Department of Orthopedic Surgery, Lyon Ortho Clinic, Le Trait d’Union 29 Bis av Sources, 69009 Lyon, France; Nashville Hip Institute, Orthopaedic Surgery, 2004 Hayes St Suite 700 Nashville, TN 37203, USA; Nashville Hip Institute, Orthopaedic Surgery, 2004 Hayes St Suite 700 Nashville, TN 37203, USA; Department of Orthopedic Surgery, The Centers for Advanced Orthopeadics, 6707 Democracy Blvd Suite 504 Bethesda, MD 20817, USA; Department of Orthopaedic Surgery, Orthopadische Chirurgie Munchen, Steinerstrabe 6, 81369 Munchen, Germany; Department of Orthopedic Surgery, OrthopedicONE, 6810 Perimeter Dr. Suite 200 Dublin, OH 43016, USA; Department of Physical Therapy, UPMC Rehabilitation Institute, 3200 S Water St. Pittsburgh, PA 15203, United States; Duquesne University, Department of Physical Therapy, 141 Health Sciences Building, 600 Forbes Avenue, Pittsburgh, PA 15282, USA; Department of Orthopedic Surgery, OrthopedicONE, 6810 Perimeter Dr. Suite 200 Dublin, OH 43016, USA; Department of Physical Therapy, Physio & More, 27 Shabtai Yaacov, Tel Aviv-Jaffa 6962806, Isreal; Department of Physical Therapy, Wichita State Universtiy, 213 N Mead St Wichita, KS 67202, USA; Department of Physical Therapy, UPMC Children’s Hospital of Pittsburgh, 4401 Penn Ave, Pittsburgh, PA 15224, USA; Department of Orthopaedic Surgery, Hip Arthroscopy Australia, Wellington Grange St Johns Hospital, 30 Cascade Road, South Hobart TAS 7004, Australia; Department of Orthopaedic Surgery, Stanford University, 450 Broadway St. Redwood City, CA 94063, USA; Australian Physiotherapy, 21 Erin Street Richmond, VIC 3121, Australia; Department of Physical Therapy, NISMAT, 210 E 64th St 5th Floor, New York, NY 10065, USA; Department of Physical Therapy, Belmont University, 1900 Belmont Blvd, Nashville, TN 37212, USA; Duquesne University, Department of Physical Therapy, 141 Health Sciences Building, 600 Forbes Avenue, Pittsburgh, PA 15282, USA

**Keywords:** physiotherapy, consensus, femoroacetabular impingement, hip arthroscopy, rehabilitation

## Abstract

The 2024 International Society for Hip Preservation physiotherapy agreement presents expert-driven rehabilitation guidelines for use following hip arthroscopy (HA) for femoroacetabular impingement syndrome (FAIS). The expert-panel consisted of 11 physiotherapists and eight surgeons representing five countries specializing hip preservation. Despite the favorable outcomes of HA for FAIS demonstrated in the current literature, there is a paucity of research supporting a specific physiotherapy protocol. These guidelines were created utilizing a modified-Delphi technique where panelists utilized the Grading of Recommendations, Assessment, Development, and Evaluation scale to determine inclusion of treatment interventions within each phase. The panelists recommend six phases of rehabilitation with a time and milestone-based approach beginning in the immediate post-operative period and continuing the through the return to sport phase. These expert-driven guidelines will help reduce practice variation and standardize post-operative timelines.

## Introduction

Femoroacetabular impingement syndrome (FAIS) is defined as premature contact between the femoral head–neck junction and the acetabulum, mostly secondary to bony abnormalities, leading to chondral injury, labral tearing, and early onset hip arthritis [1]. Initial treatment for FAIS consists of conservative rehabilitation. However, when conservative management fails to reduce pain or improve quality of life, surgical management may be recommended [2]. Hip arthroscopy (HA) is a minimally invasive surgical procedure that can correct bony abnormalities of the hip joint and address soft tissue damage including acetabular labrum tears and articular cartilage injuries [3]. HA for FAIS has demonstrated good to excellent short- and mid-term outcomes, with improvements in self-reported quality of life and pain, and some studies suggest HA may also delay the onset of hip osteoarthritis [4]. Furthermore, systematic review by Minkara *et al.* [5] reported 87.7% of patients return to sport following HA.

Restoration of pain-free range of motion (ROM), increased strength of the lumbopelvic musculature, and normalization of lower extremity (LE) neuromuscular (NM) control are commonly used during post-operative rehabilitation following HA. Despite the favorable outcomes of HA for FAIS, there is limited research supporting a specific post-operative physical therapy protocol. There are several clinical commentaries on the topic [6–8]. However wide variations in timelines, restrictions, precautions, and suggested exercises exist when comparing post-operative rehabilitation guidelines. The purpose of this modified Delphi study was to present expert-driven, multi-phase rehabilitation guidelines for use following HA for FAIS.

## Methods

### Study participants

Members of the International Society for Hip Preservation (ISHA) with expertise in rehabilitation following HA were identified by the senior author, and 100% of invited panelists (n = 18) agreed to participate. The panel consisted of 11 physiotherapists and eight surgeons specializing in hip preservation representing five countries with an average of 25.7 years (range: 11–42) of experience. A summary of the panelist attributes is presented in Table 1.

**Table 1:** Participant attributes.

Physiotherapists							
Name	Years of Experience	Specialty (i.e. sports, ortho, trauma, region of body)	Number of hip-specific consultations per week	Number of hip-specific physiotherapy patients per year	Academic post (y/n)	Actively participates in research (y/n)	ISHA member (y/n)
	14	Sports and ortho	30	250	N	Y	Y
	11	Sports and ortho; Hip and pelvis	25	250	N	Y	Y
	23	Sports and ortho; Hip and pelvis	10	100	Y	Y	Y
	25	Sports and Ortho	NA- Academia				
	17	Sports and Ortho; Hip/Pelvis	40	250	N	Y	Y
	30	Sports and Ortho; Hip and Pelvis	30	180	Y	Y	Y
	31	Sports and Ortho	3	50	Y	Y	Y
	32	Sports and Ortho	NA- Academia		Y	Y	Y
	27	Sports Medicine and Tennis	40	300	Y	Y	Y
	33	Sports and Ortho	11	56	N	Y	Y
	42	Sports and ortho; Hip and Pelvis	5	100	Y	Y	Y
**Orthopedic Surgeons**							
**Name**	**Years of experience**	**Specialty**	**Number of hip-specific consultations per week**	**Hip surgical procedures per year**	**Academic post (y/n)**	**Actively involved in research (y/n)**	**ISHA member (y/n)**
	20	Hip surgery	50	600	Y	Y	Y
	37	Ortho and sports medicine	20	400	Y	Y	Y
	24	Sports medicine and hip arthroscopy	30	150 HA	N	Y	Y
	27	Hip	40 personal +40 fellow	300 HA, 30 PAO, 10 other open preservation, 150 THA	N	Y	Y
	25	Hip Surgery	50	500	N	Y	Y
	8	Hip surgery	30	150	Y	Y	Y
	33	Arthroscopic Hip Surgery	35–40	400	Y	Y	Y
	30	Sports Medicine & Hip Arthroscopy	60	150	Y	Y	Y

### Delphi survey

This study utilized a modified-Delphi technique with methods similar to those presented in previous consensus statements [2,9,10]. In the first round of the Delphi process, two members of the panel (AD and RM) presented proposed post-operative phases including timeframes, rehabilitation domains, and goals based upon the guidelines set forth in the 2019 ISHA physiotherapy agreement on assessment and treatment of FAIS (Appendix I) [2]. The panelists agreed on the proposed post-operative phases and were given the opportunity to present a comprehensive list of interventions for each phase and rehabilitation domain for potential consideration. Two members of the panel (AD and RM) compiled the list of suggested exercises and created a proposed post-operative progression (Appendix II). Utilizing the Grading of Recommendations, Assessment, Development, and Evaluation (GRADE) scale, the panelist graded each proposed treatment intervention [11]. The GRADE scale is a 1–9-point scale based upon importance: not important for inclusion (1–3 points), important but not critical (4–6 points), critical to for inclusion (7–9 points) [11]. An intervention was included only if it was deemed critical by >70% of panelists. Interventions that deemed not important by >70% of panelists were excluded from the final guidelines and any further survey round. Consensus was achieved for all interventions presented within each phase of rehabilitation during the second survey round. Following physiotherapist consensus, the identified surgeons were given the opportunity to review and revise the presented guidelines.

## Results

All 11 (100%) physiotherapists who were deemed experts in the rehabilitation of HA for FAIS participated in each survey round and provided feedback on each item utilizing the GRADE scale. Panelists achieved 100% consensus for the proposed rehabilitation phases during the first survey round. Consensus was achieved for 100% of the items included in the guidelines by the second survey round. The panelists agreed upon a six-phase rehabilitation progression, from the immediate post-operative phase through the return-to-sport phase, as well as proposed time frames, goals, and specific treatment interventions for each phase (Table 2). All surgeons (100%) approved the proposed guidelines.

**Table 2:** Post-operative rehabilitation guidelines for use following hip arthroscopy.

Immediate Post-Operative Phase (0–2 Weeks)	
Goal: 1) *Protection of the surgical hip*	
Pain and Swelling Control	Cryotherapy
	Nature of surgical procedure
	ROM precautions
	Positional modifications
Patient Education	Stages of post-operative rehabilitation
	Return to activity timelines
	Weight bearing precautions
	Appropriate use of assistive device
	Caregiver PROM within protected range
	Circumduction PROM
	Supine AROM for knee/ankle
ROM/Flexibility	Quadriceps stretching in modified Thomas position
	Upright stationary bike
	Transverse abdominis, quadriceps, deep hip rotator, and gluteal setting
	Hip IR/ER isometrics
Strengthening	Hip ADD/ABD isometrics
	Supine lumbopelvic AROM
Progression of Weight Bearing (2–4 Weeks)	
*Goals:*	
1) *90° pain-free hip flexion*	
2) 10° hip extension	
ROM/Flexibility	Upright stationary bike
	Quadriceps stretching in prone
	Quadruped rocking (hand-to-heel rocking)
	Quadruped lumbopelvic AROM
Soft Tissue Mobilization	Psoas, rectus femoris, TFL, hip adductors, gluteals, and ITB
Strengthening	Standing hip ABD
	Initiation of bridge progression
	Quadruped hip extension and ABD
	Initiation of TA progression in supine and quadruped
Neuromuscular Control	Weight shifting
	Heel-toe-raise
Initiation of Strengthening Exercise (4–8 Weeks)	
*Goals:*	
1) *Normal, symmetrical ROM*	
2) *Normalized gait pattern without use of AD*	
ROM/Flexibility	Upright stationary bike
	Kneeling hip flexor stretch
	Initiate FABER AROM
	Supine ADD stretching
Soft Tissue Mobilization	As indicated to restore hip ROM and flexibility
Cardiovascular Endurance	Walking progression
Strengthening	Deep hip rotator progression^a^
	Bridge progression
	Side-lying hip abduction progression^a^
	Forward and side plank hip progression
	Seated knee extension
	Body weight squats to 90°
	Supine, quadruped, and tall kneel TA progression
Neuromuscular Control	Quadruped lumbopelvic stability drills
	Single leg static balance
	Initiate double to single leg squat progression
Early Functional Restoration (Weeks 8–12)	
*Goals:*	
1) *Appropriate pelvic control during single leg activities*	
Cardiovascular Endurance	Stationary bike with progressive resistance
	Elliptical
	Swimming with pool buoy between legs
Flexibility	End-range stretching
	Initiation of yoga activities
Strengthening and Neuromuscular Control	Weighted double leg squats
	Double and single leg dead lifts
	Lunges
	Single leg step downs
	Single leg squats
	Dynamic balance drills
Late Functional Restoration (Weeks 12–18)	
*Goals:*	
1) *Strength > 80% of contralateral side*	
2) *Score > 85% on Hip Outcome Score – Sport Subscale*	
3) *80% LSI during Star Excursion Balance Test*	
Cardiovascular Endurance	Straight line running progression
	Swimming with initiation of kicking
Strengthening and Neuromuscular Control	Single leg rotational control exercises
	Low intensity agility and plyometric progression
Return to Sport (Weeks 18+)	
*Goals:*	
1) *Appropriate pelvic and lower extremity control during all sport specific activities*	
Sport-Specific Training	Agility and plyometric progression
	Sport specific exercise
	Sprinting

Abbreviations: PROM, passive range of motion; AROM, active range of motion; IR, internal rotation; ER, external rotation; ADD, adductor; ABD, abductor; TFL, tensor fasca lata; ITB, iliotibial band; TA, transverse abdominis; LSI, limb symmetry index.

a with resistance as tolerated.

## Discussion

The purpose of this consensus statement is to present expert-driven rehabilitation guidelines for use following HA for FAIS, addressing a critical gap in standardizing post-operative care. The panelists agreed upon a six-phase rehabilitation progression from the immediate post-operative phase through the return-to-sport phase. In the absence of a well-accepted rehabilitation protocol following HA for FAIS, these guidelines can be utilized to reduce practice variation to ensure consistent outcomes among this population.

HA for FAIS has consistently demonstrated improvements in hip function, quality of life, and pain in both short and mid-long-term outcomes. A 2023 systematic review by Jan *et al.* [12] reported individuals undergoing primary HA for FAIS demonstrated significant improvement 5-years post-operatively. In addition to improvements in patient reported outcomes, return to sport rates further illustrate the effectiveness of HA for FAIS. Weber *et al.* [13] reported 89.7% of Division I student-athletes return to sport at a similar rate except endurance athletes who return at a lower rate of 66.6%. More recently, Lucenti *et al.* [14] found 79.6% of individuals returned to sport at the same or higher level at an average of 14.3 months following HA for FAIS. While variations exist depending individual goals and sporting demands, these results collectively highlight the effectiveness of HA for FAIS.

To achieve optimal outcomes, including return to prior level of sport participation, it is well accepted that formal post-operative physiotherapy is essential. Evidence indicates that improved strength and ROM measures post-operatively are linked to quality of life. At 1–2 years following HA for chondrolabral pathology, Kemp *et al.* [15] reported hip flexion ROM and adduction strength were associated with improved quality of life. Similarly, Ishoi *et al.* [16] reported higher maximal hip extension strength was significantly associated with increased ability to participate fully at prior level of sport participation. Furthermore, while symptoms, function, and satisfaction have been shown to improve as early as 14 weeks, hip strength measures lag and continue to improve for up to a year post-operatively [17]. Adequate strength also plays a role in achieving appropriate pelvic and lower extremity neuromuscular control during single leg tasks. In a cross sectional study of individuals who had undergone HA, Freke *et al.* [18] reported Star Excursion Balance Test reach values were decreased in all directions at both 3- and 6- months compared to healthy controls, except for posterolateral which improved to the level of healthy controls at 6-months. These studies highlight the importance of structured PT following HA for FAIS throughout the entirety of recovery. While multi-phase PT protocols exist in the literature, there is a wide variety of timelines, post-operative precautions, and exercise progressions leading to varying practice patterns in this population [6].

The presented-expert driven guidelines include a six-phase rehabilitation progression, including temporal and functional based progression. Proposed time frames, goals, and specific treatment interventions for each phase are presented in Table 2. It should be noted implementation of these guidelines in clinical practice requires approval from the referring surgeon and deviations may be appropriated based individual patient characteristics and surgical procedure performed.

### Phase One: Immediate Post-Operative Phase (0–2 Weeks)

During Phase One, there is an emphasis on pain and swelling control, patient education, gentle ROM and flexibility exercises, and submaximal isometric activation exercises. Flat foot weight bearing with the appropriate assistive device (AD) is recommended to minimize overactivity of the iliopsoas. Additionally, gentle stretching of the quadriceps can be initiated in a modified Thomas position maintaining neutral hip extension to protect the anterior hip capsule.

### Phase Two: Progression of Weight Bearing (2–4 Weeks)

During Phase Two, individuals begin to wean off an AD and begin light double leg standing exercise, including weight shifting, mini squats, and heel raises. Along with use of an upright stationary bike, ROM into hip flexion can be safely progressed in the quadruped position, protecting the healing structures of the anterior hip joint. By the end of phase two, individuals should be comfortable ambulating short distances with a normalized gait pattern.

### Phase Three: Initiation of Strengthening Exercise (4–8 Weeks)

During Phase Three, an emphasis is placed upon progression of double leg weight bearing exercises targeting the lumbopelvic stabilizers. When appropriate, progression to gentle single leg strengthening may begin focusing on static control of the lumbopelvic and lower extremity. A walking program may also be initiated during this phase to improve tolerance to joint loading as well as to facilitate light cardiovascular exercise. Prior to initiating a walking program, clinicians should ensure hip extension ROM and anterior hip flexibility is normalized to allow for terminal hip extension during the stance phase.

### Phase Four: Early Functional Restoration (Weeks 8–12)

During Phase Four, rehabilitation should focus on dynamic, multi-planar single leg control training. Swimming with a pool buoy between the lower extremities can be initiated to further encourage cardiovascular endurance. Kicking while swimming is not recommended during this phase to continue to protect the iliopsoas. While foundational strengthening is still encouraged, a shift to NM control drills is recommended in preparation for initiation of a return-to-sport progression. The panelists also recommend monitoring fear of movement utilizing the Tampa Scale of Kinesiophobia, with a score >37 indicating significant kinesiophobia [19]. Browning *et al.* [20] reported a negative correlation between kinesiophobia and return to sport rates at 1-year following HA.

### Phase Five: Late Functional Restoration and Initiation of Impact (Weeks 12–18)

During Phase Five, individuals may begin a straight-line running program if they are demonstrating appropriate single leg NM control tasks, such as lateral step downs or single leg squats, demonstrating strength >80% of uninvolved side, and score >85% on the Hip Outcome Score-Sport Specific Scale. Straight line running should be progressed gradually, focusing on tolerance to light intervals before progressing speed and distance. When individuals are demonstrating good tolerance to running, low level agility drills, such as ladder drills, and light plyometrics can be initiated. These exercises should be progressed based on joint irritability and pelvic and lower extremity control. Gentle kicking during swimming may also be initiated. During this phase, specific work demands, such as heavy lifting, should also be addressed.

### Phase Six: Return-to-Sport Phase (Weeks 18+)

In those who wish to return to sport, continuation of structured PT is recommended in order to ensure safe participation. During the final phase, high level agility drills involving cutting and pivoting as well as advanced plyometrics should be initiated based on individual sporting demands. Like phase five, these exercises should be progressed based on irritability and pelvic and lower extremity control. When appropriate, sport specific movements should be incorporated to ensure readiness for individual sporting demands. By the end of this phase, individuals should be demonstrating excellent tolerance and NM control during all sport-specific tasks.

There are several limitations to the proposed rehabilitation guidelines. These guidelines do not consider the variety of procedures that may be performed during HA, including but not limited to the use of biologics, microfracture or cartilage repair, and capsular closure, or revision surgeries. Revision surgeries, which may account for 10% of primary HA procedures, have been shown to demonstrate poorer outcomes than primary HA for FAIS [21]. Additionally, the presence of concurrent structural abnormalities such as borderline hip dysplasia should also be considered. Clinicians should utilize these recommendations as guidelines, considering any precautions that may slow progression though the proposed phases for each individual patient. Additionally, these guidelines are based upon expert-opinions. Future studies are needed to assess patient outcomes with the use of these guidelines. Additionally, these guidelines do not expand upon the return-to-sport progression. Future guidelines are needed to present expert-based and evidence driven recommendations on progression from return-to-run to return-to-sport following HA for FAIS.

## Conclusion

This consensus presents a six-phase rehabilitation progression for use in individuals following HA for FAIS. These expert-driven guidelines will help reduce practice variation and standardize post-operative timelines. Future studies need to focus on patient reported outcome measures to confirm the guidelines presented in this current study as well as variations that may exist due to specific surgical procedure performed during HA.

## Supplementary Material

HA_for_FAIS_consensus_Appendix_7_9_25_hnaf055

## Data Availability

The data generated and analyzed during the current study are available from the corresponding author upon request.
