# Supplementary material for: The 2024 international society for hip preservation physiotherapy agreement on post-operative rehabilitation following hip arthroscopy for femoroacetabular impingement syndrome
Source: J Hip Preserv Surg. 2025 Oct 27;13(3):288–94. doi: 10.1093/jhps/hnaf055 (PMC13653055; doi:10.1093/jhps/hnaf055)
Supplement: Supplementary file 1 [file ha_for_fais_consensus_appendix_7_9_25_hnaf055.docx]

**APPENDIX I: Initial proposed post-operative time frames and goals**

| **Phase** | **Length of Phase** | **Category of Interventions** | **Goals** |
| --- | --- | --- | --- |
| **Immediate Post-Operative Period** | 0-2 Weeks | Pain and swelling control | Protection of surgical hip |
|  |  | Patient education |  |
|  |  | ROM/Flexibility |  |
|  |  | Strengthening |  |
| **Early Post-Operative Period** | 2-4 Weeks | Pain and swelling control | 90 degrees hip flexion |
|  |  | ROM/Flexibility | 10 degrees hip extension |
|  |  | Soft tissue mobilization |  |
|  |  | Strengthening |  |
|  |  | Neuromuscular control |  |
| **Initiation of Strengthening** | 4-8 Weeks | ROM/Flexibility | Normal, symmetrical ROM |
|  |  | Soft tissue mobilization | Normalized gait pattern without use of AD |
|  |  | Cardiovascular exercise |  |
|  |  | Strengthening |  |
|  |  | Neuromuscular control |  |
| **Early Functional Restoration** | 8-12 Weeks | Cardiovascular exercise | Appropriate pelvic control during single leg activities |
|  |  | Flexibility |  |
|  |  | Strengthening and neuromuscular control |  |
| **Late Functional Restoration** | 12-18 Weeks | Cardiovascular exercise | Strength >80% of contralateral side |
|  |  | Strengthening and neuromuscular control | Score >85% on HOS-sport subscale |
|  |  |  | 80% LSI during SEBT |
| **Return to Sport** | 18+ Weeks | Sport specific training | appropriate pelvic and lower extremity neuromuscular control during all sport-specific activities |

**APPENDIX II: Initial proposed post-operative interventions**

| **Phase of Rehab (length)** | **Category** | **Intervention** | **Not Important (Do not need to include)**  **1-3 points** | **Somewhat Important (May Include)**  **4-6 points** | **Important (Should include)**  **7-9 points** |
| --- | --- | --- | --- | --- | --- |
| **Immediate Post-Operative (0-2 Weeks)**  Goals: Protection of surgical hip | Pain and Swelling Control | Cryotherapy |  |  |  |
|  |  | Brace |  |  |  |
|  |  | E-Stim |  |  |  |
|  | Pt Education | Nature of surgery |  |  |  |
|  |  | ROM Precautions |  |  |  |
|  |  | Positional modification |  |  |  |
|  |  | Rehabilitation stages |  |  |  |
|  |  | Return to activity timelines |  |  |  |
|  |  | Gait training/use of AD |  |  |  |
|  | ROM/Flexibility | Caregiver PROM within protected range |  |  |  |
|  |  | Circumduction PROM |  |  |  |
|  |  | Supine AROM at knee/ankle  Prone lying as tolerated |  |  |  |
|  |  | Quadriceps stretching |  |  |  |
|  |  | Pain-free hand to heel rock (quadruped rocking) |  |  |  |
|  |  | Upright stationary bike |  |  |  |
|  |  | CPM Machine |  |  |  |
|  | Strengthening | Abdominal, quad, gluteal setting and activation |  |  |  |
|  |  | Supine lumbopelvic AROM |  |  |  |
|  |  | Pain-free hip isometrics (ABD, ADD, IR/ER) |  |  |  |
|  |  | Concentric hip strengthening |  |  |  |
| **Early Post-Operative Phase (2-4 Weeks)**  Goals: 90 degrees pain-free flexion  10 degrees hip extension | Pain and Swelling Control | Cryotherapy |  |  |  |
|  |  | Brace |  |  |  |
|  |  | E-Stim |  |  |  |
|  | ROM/Flexibility | Upright stationary bike |  |  |  |
|  |  | Quadriceps stretching in prone |  |  |  |
|  |  | Quadruped lumbopelvic AROM |  |  |  |
|  |  | Quadruped rocking (hand-to-heel rocking) |  |  |  |
|  | Soft Tissue Mobilization | Psoas, rectus femoris, TFL, ADD, gluteals, ITB |  |  |  |
|  | Strengthening | Bridging |  |  |  |
|  |  | Standing hip ABD |  |  |  |
|  |  | Sidelying ABD progression |  |  |  |
|  |  | Supine TA progression |  |  |  |
|  |  | Quadruped hip ABD and EXT |  |  |  |
|  | Neuromuscular control | Weight shifting |  |  |  |
|  |  | Heel-toe raise |  |  |  |
| **Initiation of Strengthening Exercise (4-8 weeks)**  Goals: Normal, symmetrical ROM  Normalized gait pattern without use of AD | ROM/Flexibility | Upright stationary bike |  |  |  |
|  |  | FABER AROM |  |  |  |
|  |  | Kneeling hip flexor stretch |  |  |  |
|  |  | Supine ADD stretching |  |  |  |
|  |  | Joint mobilizations |  |  |  |
|  | Soft-tissue mobilization | Psoas, rectus femoris, TFL, ADD, gluteals, ITB |  |  |  |
|  | Cardiovascular exercise | Walking progression |  |  |  |
|  | Strengthening | Deep hip rotator progression with resistance as tolerated |  |  |  |
|  |  | Bridge progression |  |  |  |
|  |  | Prone hip extension |  |  |  |
|  |  | Sidelying hip ABD with resistance as tolerated |  |  |  |
|  |  | Leg press |  |  |  |
|  |  | Seated hamstring curl |  |  |  |
|  |  | Bodyweight squats |  |  |  |
|  |  | TA progression into quadruped and tall kneel |  |  |  |
|  |  | Plank/side plank progression |  |  |  |
|  |  | Step downs |  |  |  |
|  | Neuromuscular Control | Quadruped, tall kneel, half-kneel and standing lumbopelvic stability drills |  |  |  |
|  |  | Single leg stance with progression as tolerated |  |  |  |
|  |  | Single leg balance |  |  |  |
|  |  | Double to single leg squat progression |  |  |  |
| **Early Functional Restoration (weeks 8-12)**  Goals: appropriate pelvic control during single leg activities | Cardiovascular Exercise | Upright stationary bike with progressive resistance |  |  |  |
|  |  | Walking progression |  |  |  |
|  |  | Swimming with pool buoy between legs |  |  |  |
|  | Flexibility | End range stretching |  |  |  |
|  | Strengthening and Neuromuscular control | Weighted double leg squats |  |  |  |
|  |  | Double and single leg dead lifts |  |  |  |
|  |  | Lunges |  |  |  |
|  |  | Single leg step downs |  |  |  |
|  |  | Single leg squats |  |  |  |
|  |  | Dynamic balance drills |  |  |  |
| **Late Functional Restoration (Weeks 12-18)**  Goals: Strength >80% of contralateral side  Score >85% on HOS- sport specific subscale  80% LSI during SEBT | Cardiovascular Exercise | Straight line running progression |  |  |  |
|  |  | Swimming with initiation of kicking |  |  |  |
|  | Strengthening and Neuromuscular control | Single leg rotational control exercises |  |  |  |
|  |  | Low intensity agility and plyometric progression |  |  |  |
| **Return to Sport (weeks 18+)**  Goal: appropriate pelvic and lower extremity neuromuscular control during all sport-specific activities | Sport Specific Training | Agility and plyometric progression |  |  |  |
|  |  | Sport specific exercise |  |  |  |
|  |  | Sprinting |  |  |  |
